# Dataset on the knowledge, attitude and practices of biomedical wastes management among Neyshabur hospital’s healthcare personnel

**DOI:** 10.1016/j.dib.2018.02.024

**Published:** 2018-02-15

**Authors:** Mahmood Alimohammadi, Mahmood Yousefi, Fatemeh Azizi Mayvan, Vahid Taghavimanesh, Hossein Navai, Ali Akbar Mohammadi

**Affiliations:** a Department of Environmental Health Engineering, School of Public Health, Tehran University of Medical Sciences, Tehran, Iran; b Center for Water Quality Research (CWQR), Institute for Environmental Research (IER), Tehran University of Medical Sciences, Tehran, Iran; c Master of Biostatistics, Neyshabur University of Medical Sciences, Neyshabur, Iran; d Department of Environmental Health Engineering, School of Public Health, Mashhad University of Medical Sciences, Mashhad, Iran; e Students Research Committee, Neyshabur University of Medical Sciences, Neyshabur, Iran; f Department of Environmental Health Engineering, Neyshabur University of Medical Sciences, Neyshabur, Iran

**Keywords:** Bio-medical waste, Health care worker, Knowledge, Awareness, Attitude

## Abstract

The data presented in this article are related to the research article entitled “knowledge, attitude and performance regarding waste management among the HCWs in hospitals affiliated with the Neyshabur City, Iran”. A researcher-made questionnaire (accessible as an attachment) containing 4 parts of demographic information, knowledge (24 questions), attitude (6 questions) and practices (6 questions) was used for data gathering. Kruskal- Wallis test, Mann-Whitney U and Spearman correlation coefficient were used to analyze the data. The significance level was set at 0.05 for the test. Data Analyzing showed the relationship between attitude and Practices with a correlation coefficient of 0.177 was statistically significant (*P* = 0.01). Also, according to this research, the relationship between the individuals' work experience with knowledge, attitude, and Practices with their correlation coefficients of 0.178, 0.247, and 0.152, respectively were significant (*P* = 0.018, *P* = 0.001, *P* = 0.043). Furthermore, the relationship between age with knowledge and practice was not significant (*P* = 0.605 and *P* = 0.102, respectively) and its relationship with attitude was significant with a correlation coefficient of 0.154 (*P* = 0.028).

**Specifications table**

**Table:** 

Subject area	Neyshabur Hospital's Healthcare Personnel
More specific subject area	Describe narrower subject area
Type of data	Tables
How data was acquired	The instrument used in this study was a researcher-made questionnaire consisted of four sections. A researcher-made questionnaire (accessible as an attachment) containing 4 parts of general information, knowledge (24 questions), attitude (6 questions) and practices (6 questions) was used for data gathering. Its face and content validity were confirmed by relevant experts while the reliability of the questionnaire was determined by Kappa Test-Retest. The kappa was determined 0.75, 0.73 and 0.75, for knowledge, attitude and practice, respectively.
Data format	Raw, Analyzed
Experimental factors	The mentioned parameters above, in abstract section, were analyzed according to the Completed questionnaires.
Experimental features	The levels of knowledge, attitude and practices of biomedical wastes management among Neyshabur hospital's healthcare personnel were determined.
Data source location	Neyshabur, Razavi Khorasan province, Iran.
Data accessibility	The data are available whit this article

**Value of the data**

• Mismanagement of the biomedical waste can result in environmental and occupational health risks.
• Education is one of the essential and important components in the field of waste management.
• Staff training will improve the policies and procedures of managing hospital waste
• Outcomes of statistical analyses from this study, suggested to hold some periods of compulsory and effective education with partnership of university for hospital in the field general waste management hospital.

## 1 Data

Table 1 Shows descriptive statistics related to the demographic information of the HCW and also Table 2, Table 3 shows respectively Spearman correlation coefficients between knowledge, attitude, practices, age, and work experience of the individuals and average of knowledge, attitude, and Practices among different jobs.

**Table 1:** Descriptive statistics related to the demographic information of the HCW.

**Variables**	**Frequency (%)**
**Gender**	
Male	**75(35.9)**
Female	**134(64.1)**
**Age**	
Max	**60**
Min	**21**
Mean	**23 ± 31**
**Working status**	
Doctor	**10(4.8)**
Nurses	**138(66)**
Radiologist	**14(6.7)**
Paramedics	**44(22.1)**
Total	**209(100)**

**Table 2:** Spearman correlation coefficients between knowledge, attitude, practices, age, and work experience of the individuals.

	**Knowledge**	**Attitude**	**Practices**	**Age**	Work experience
**Knowledge**	1	0	0	0	**0**
**Attitude**	−0.016	1	0	0	**0**
	(*P* = 0.823)				
**Practices**	0.133	0.177		0	**0**
	(*P* = 0.055)	(*P* = 0.010)⁎	1		
**Age**	0.037	0.154	0.115		
	(*P* = 0.605)	(*P* = 0.028)⁎	(*P* = 0.102)	1	
**Work experience**	0.178	0.247	0.152	0.183	
	(*P* = 0.018)⁎	(*P* = 0.001)⁎	(*P* = 0.043)⁎	(*P* < 0.0001)⁎	1

⁎ *P* = 0.05.

**Table 3:** Average of knowledge, attitude, and practices among different jobs.

**Job**	**Knowledge**	**Attitude**	**Practices**
Nurses	4.49 ± 21.75	0.85 ± 5.06	0.93 ± 5.17
Paramedics	4.76 ± 20.48	0.80 ± 5.47	0.61 ± 5.36
Radiologists	4.98 ± 22.94	0.77 ± 5.14	1.18 ± 5.00
Doctors	2.64 ± 21.90	1.08 ± 5.10	0.63 ± 4.20

## 2 Materials and methods

The present research was a cross-sectional and descriptive-analytic study which was conducted in 2015 to assess the knowledge, attitude and Practices regarding waste management among the HCWs in hospitals affiliated with the Neyshabur Faculty of Medical Sciences. In this study, all personnel working in the wards of Hakim Hospital and Bahman 22nd Hospital, including doctors, nurses, paramedics, and service personnel participated in the study. The instrument used in this study was a researcher-made questionnaire consisted of four sections. A researcher-made questionnaire (accessible as an attachment) containing 4 parts of general information, knowledge (24 questions), attitude (6 questions) and practices (6 questions) was used for data gathering. Its face and content validity were confirmed by relevant experts while the reliability of the questionnaire was determined by Kappa Test-Retest. The kappa was determined 0.75, 0.73 and 0.75, for knowledge, attitude and practice, respectively.

The number of items to assess knowledge was 24, and to calculate the score of knowledge, each correct answer was awarded 2 points and for wrong answers and “I do not know”, zero point and one point was considered, respectively. Therefore, the knowledge score was placed between zero (no correct answers) to 48 (the correct answer to all questions). Six questions were asked in order to measure the attitude and Practices of each participant; and to calculate the score for the assessment and practices, if they chose a desirable and expected Practices, 1 point and regarding the other functions, zero point were considered. On this basis, the score of assessment and Practices were considered between zero (poor Practices in all cases considered) to 6 (excellent Practices in all cases considered) [1], [2], [3], [4], [5], [6], [7], [8], [9], [10], [11]. After collecting the questionnaires, the data entered in the computer and data was analyzed using software of SPSS 17. Since the Kolmogorov- Smirnov test didn't show a normal distribution of grades for knowledge and Practices, Kruskal- Wallis test, Mann-Whitney U and Spearman correlation coefficient were used to analyze the data. The significance level was set at 0.05 for the test.

## References

[1] Amouei A., Tahmasbizadeh M., Asgharnia H., Fallah H., Mohammadi A. (2012). Quantity and quality of solid wastes in the hospitals of Babol university of medical sciences, North of Iran. J. Appl. Sci. Environ. Sanit..

[2] Nabizadeh R., Faraji H., Mohammadi A. (2014). Solid waste production and its management in dental clinics in Gorgan, Northern Iran. Int. J. Occup. Environ. Med..

[3] Dehghani M.H., Omrani G.A., Nadafi K., Marosi M., Azam K. (2011). Solid waste management in physicians' offices in Sabzevar. Hakim Res. J..

[4] Ghanami Z., Amouei A., Fallah H., Asgharnia H., Mohammadi A., Naghipour D. (2013). Survey of qualitative and quantitative characteristics of municipal solid wastes in North of Iran (Babolsar city) in 2012. Health Scope.

[5] Safdari M., Mirzaei Alavijeh M., Ehrampoosh M.H., Ghaneian M.T., Morowatisharifabad M.A. (2013). Knowledge, attitude and practices students of shahid sadoughi university of medical sciences-yazd about recycling solid material: a short report. Rafsanjan Univ. Med. Sci. J..

[6] Rabeie O.L., Miranzadeh M.B., Fallah S.H., Dehqan S., Moulana Z., Amouei A. (2012). Determination of hospital waste composition and management in Amol city, Iran. Health Scope.

[7] Mahvi A.H., Roodbari A.A., Nodehi R.N., Nasseri S., Dehghani M.H., Alimohammadi M. (2012). Improvement of landfill leachate biodegradability with ultrasonic process. PloS One.

[8] Amouei A., Miranzadeh M.B., Asgharnia H., Fallah H., Mohammadi A. (2012). Study of quality and quantity of clinical laboratories solid wastes in North of Iran. J. Appl. Technol. Environ. Sanit..

[9] Asgharnia H., Tirgar A., Amouei A., Fallah S., Khafri S., Mohammadi A. (2014). Noise pollution in the teaching hospitals of Babol (Iran) in 2012. J. Babol Univ. Med. Sci..

[10] Ehrampoosh M.H., Parsa A., Kiani G., Iraji F., Rezaei A. (2012). Knowledge, attitude and practices toward solid waste management among the residents of Ramshe, Iran. J. Health Syst. Res..

[11] Gbèblonoudo Dassou A., Carval D., Dépigny S., Fansi G., Tixier P. (2016). Dataset on the abundance of ants and Cosmopolites sordidus damage in plantain fields with intercropped plants. Data Brief.

